# Anti-Inflammatory Effects of *Caulerpa okamurae* Extracts on *Porphyromonas gingivalis*-Stimulated RAW 264.7 Macrophages

**DOI:** 10.3390/cimb47060388

**Published:** 2025-05-23

**Authors:** Chae-yun Lee, Min-jeong Kim, Hyun-jin Kim

**Affiliations:** 1 Department of Convergence Technology for Food Industry, Wonkwang University, Iksan 54538, Republic of Korea; 14_1210@naver.com (C.-y.L.); lucklove519@naver.com (M.-j.K.); 2 Institute of Biomaterial Implant, Department of Oral Anatomy, School of Dentistry, Wonkwang University, Iksan 54538, Republic of Korea

**Keywords:** *Caulepra okamurae*, *Porphyromonas gingivalis*, anti-inflammation, periodontitis, NF-κB, macrophage

## Abstract

*Caulebra okamurae* (*C. okamurae*), a green seaweed, has been reported to exhibit pharmacological properties, including anti-obesity and anti-diabetic effects. This study investigated the anti-inflammatory effects of *C. okamurae* extracts on periodontal health. The cell viability of RAW 264.7 macrophages was dose-dependently assessed using an MTS assay. The anti-inflammatory activity of *C. okamurae* on *Porphyromonas gingivalis* (*P. gingivalis*)-stimulated RAW 264.7 macrophages was evaluated by measuring nitric oxide (NO) production. mRNA expression levels of *tumor necrosis factor* (*TNF*)-*α*, *interleukin* (*IL*)-*6*, and *IL-1β* were quantified via quantitative real-time PCR (qRT-PCR). The protein expression of iNOS, p-IKKα/β, p-IκBα, and NF-κB p65 was examined using Western blot and immunofluorescence. The results demonstrated that *C. okamurae* extracts exhibited no cytotoxicity in RAW 264.7 macrophages at concentrations of 0.2, 2, 20, and 200 μg/mL. The extracts dose-dependently reduced NO production, downregulated mRNA levels of proinflammatory cytokines, and inhibited iNOS expression in *P. gingivalis*-stimulated RAW 264.7 macrophages, a model commonly used to study periodontal inflammation. Furthermore, the extracts suppressed the phosphorylation of IKKα/β and IκBα and prevented the NF-κB p65 nuclear translocation. These findings suggest that *C. okamurae* extracts inhibit NF-κB signaling activation triggered by the periodontal pathogen, highlighting their potential anti-inflammatory effects, relevant to periodontal disease.

## 1. Introduction

Periodontitis, commonly known as gum disease, is a chronic inflammatory condition characterized by the destruction of the tissues supporting the teeth and gums. The disease begins with the accumulation of plaque, a sticky film formed by bacteria on the tooth surface. If plaque is not removed and continues to accumulate, bacteria multiply, causing infection, which can eventually lead to the formation of gum pockets. These pockets create deep spaces between the gums and teeth, providing an easier pathway for bacterial invasion. As the disease progresses, it can result in alveolar bone resorption and tooth loss [1,2,3]. A key pathogen involved in the initiation and progression of periodontitis is the Gram-negative anaerobic bacterium *Porphyromonas gingivalis* (*P. gingivalis*). This bacterium adheres to the surfaces of the teeth and gums to form biofilms, which promote plaque and calculus growth, thus enhancing its pathogenicity. Notably, *P. gingivalis* produces a variety of virulent factors and enzymes that cause both direct and indirect damage to periodontal tissue. These virulent factors include lipopolysaccharide (LPS), fimbriae, cysteine proteases (e.g., gingipains), and outer membrane vesicles [4,5,6,7]. LPS overstimulates the host immune system, triggering an inflammatory response, while fimbriae facilitate bacterial attachment and invasion of host tissues. Additionally, proteases accelerate tissue damage by degrading the extracellular matrix and host proteins. These virulent factors exacerbate gingival tissue inflammation and disrupt the host immune defense system, promoting the progression of periodontitis. Thus, *P. gingivalis* acts not only as a pathogen but also plays a role in activating other pathogenic bacteria within the oral microbiome. For these reasons, *P. gingivalis* is regarded as a major pathogen and a central player in the pathogenesis of periodontitis.

Macrophages are crucial components of the innate immune system and are considered pivotal in regulating inflammatory responses and the progression of disease. In periodontitis, macrophages are recruited to the periodontal tissues in response to bacterial infection, including *Porphyromonas gingivalis*, and play a dual role. They can exert protective effects by clearing pathogens and promoting tissue repair; however, their excessive activation leads to the overproduction of proinflammatory cytokines and enzymes, contributing to tissue destruction and alveolar bone loss [8,9]. Infection by periodontal pathogens activates macrophages via Toll-like receptor 4 (TLR-4), which subsequently activates inflammatory signaling pathways, including the nuclear factor kappa B (NF-κB) pathway. Activation of NF-κB leads to the overproduction of inflammatory mediators and cytokines, accelerating tissue damage and contributing to the development of chronic inflammation [10,11,12]. The NF-κB signaling pathway plays a central role in regulating the expression of key inflammatory cytokines such as *tumor necrosis factor-alpha* (*TNF-α*), *interleukin (IL)-1β*, and *IL-6*. It also induces the expression of inducible nitric oxide synthase (iNOS), resulting in the overproduction of nitric oxide (NO) during inflammation [13,14,15].

NF-κB resides in the cytoplasm in an inactive state, bound to the IκB protein. Upon inflammatory stimulation, the IκB kinase (IKK) complex (comprising IKKα and IKKβ) phosphorylates IκBα, leading to its degradation via the proteasome pathway [16,17,18,19]. Once IκBα is degraded, NF-κB is activated and translocates to the nucleus, where it drives the expression of inflammatory cytokines, chemokines, anti-apoptotic factors, and enzymes involved in inflammation [20]. This cascade of events results in tissue damage and immune hyperactivation, contributing to the pathological progression of chronic inflammatory diseases. The NF-κB signaling pathway is a well-established amplifier of inflammatory responses, and its persistent activation is a pathological hallmark of chronic inflammatory diseases, including periodontitis [21]. Therefore, inhibiting NF-κB activation or blocking this signaling pathway is considered a promising strategy for developing anti-inflammatory therapeutic interventions.

Inflammation is a physiological response to external stimuli, infection, or tissue damage, and is an essential component of the body’s defense mechanisms [22,23,24,25]. Nonsteroidal anti-inflammatory drugs (NSAIDs) and corticosteroids are commonly used in clinical practice to suppress inflammation during the early stages of disease [26]. However, prolonged use of these drugs increases treatment costs and can lead to various side effects, including nausea, thrombocytopenia, and leukopenia [27,28]. Therefore, there has been growing interest in alternative treatments that can effectively manage inflammation while minimizing side effects. Anti-inflammatory agents derived from natural sources are gaining attention due to their potential to provide both safety and efficacy, making them valuable alternatives.

Marine organisms, especially seaweed, are abundant in bioactive compounds with anti-inflammatory, antibacterial, antiviral, and anticancer properties, which makes them of significant interest in the biomedical field [29,30]. Among them, *Caulerpa okamurae* (*C. okamurae*) is a representative seaweed species within the genus *Caulerpa*, widely distributed in the Indo-Pacific marine ecosystem, particularly in tropical and subtropical regions [31]. Recent studies have identified that extracts from *C. okamurae* contain key compounds that exhibit anti-obesity and anti-diabetic effects. Notably, *C. okamurae* has been shown to regulate key enzymes involved in lipid metabolism, promoting weight loss and lowering blood glucose levels [32]. Consequently, *C. okamurae* has emerged as a promising natural compound for the prevention and treatment of metabolic diseases such as obesity and type 2 diabetes [33]. The anti-obesity effects of *C. okamurae* are primarily mediated through pathways that inhibit adipocyte differentiation and lipid accumulation. Previous studies have demonstrated that *C. okamurae* extracts can reduce body fat accumulation and induce physiological changes associated with fat loss by modulating the expression of adipokines and lipid-related enzymes. Additionally, its anti-diabetic effects are mediated by mechanisms that improve insulin sensitivity and normalize glucose metabolism, suggesting that it may contribute to stabilizing blood glucose levels in addition to enhancing insulin secretion. *C. okamurae* extracts are believed to have potent anti-inflammatory properties. By suppressing the expression of inflammatory cytokines and inhibiting key inflammatory pathways such as NF-κB, *C. okamurae* may help control inflammation, thereby contributing to the prevention and treatment of inflammatory diseases associated with obesity and diabetes [34]. This suggests that *C. okamurae* may not only have therapeutic potential in metabolic diseases but also possess the capacity to modulate systemic inflammatory responses.

So far, the anti-inflammatory effects of *C. okamurae* in periodontitis have not been explored. This study aims to investigate whether *C. okamurae* extract exhibits anti-inflammatory activity in RAW 264.7 macrophages stimulated by *P. gingivalis* by inhibiting the NF-κB signaling pathway and suppressing proinflammatory cytokine production. The findings of this study are expected to provide a scientific basis for the potential use of *C. okamurae* extract as an anti-inflammatory agent for periodontal disease.

## 2. Material and Methods

### 2.1. Chemicals and Reagents

The CellTiter 96 AQueous One Solution Cell Proliferation Assay and Griess Reagent System were purchased from Promega (Madison, WI, USA). *IL-1β*, *IL-6*, *TNF-α*, and *β-actin* oligonucleotide primers were purchased from Bioneer (Daejeon, Republic of Korea). Antibodies targeting iNOS, phosphorylated IκB kinase (IKK)α/β (p-IKKα/β), phosphorylated NF-κB inhibitor α (IκBα) (p-IκBα), IκBα, and NF-κB (p65 subunit), as well as horseradish peroxidase (HRP)-linked anti-rabbit IgG, were purchased from Cell Signaling Technologies (Danvers, MA, USA). Antibodies targeting β-actin were purchased from Sigma-Aldrich (St. Louis, MO, USA). Antibodies targeting propidium iodide (PI) were purchased from Santa Cruz Biotechnology (Dallas, TX, USA). *P. gingivalis* was provided by OraTix Inc. (Seoul, Republic of Korea).

### 2.2. C. okamurae Extract

The *C. okamurae* extract analyzed in this study was provided by Professor Han-Gil Choi from the Department of Biological Sciences at Wonkwang University. In June 2021, samples of *C. okamurae* were gathered from Cheongsapo, Busan (35°09′ N, 129°11′ E) and transported to the laboratory in refrigerated containers designed to block light exposure. The samples were thoroughly rinsed under running tap water to eliminate impurities and then air-dried at room temperature for 10 days. The air-dried material was ground into smaller particles using a blender, followed by extraction with 94% ethanol at a sample-to-solvent ratio of 1:10. Each extraction cycle lasted 3 days, and the procedure was repeated three times to enhance yield. The collected extracts were concentrated with a rotary vacuum evaporator (Eyela N-1000, Tokyo, Japan), freeze-dried using a lyophilizer (Ilshin TFD5505, Dongducheon, Republic of Korea), and finally pulverized using a grinder (Hanil HMF1000A, Wonju, Republic of Korea). The resulting powder was stored at −70 °C in an ultra-low-temperature freezer until required for experiments.

### 2.3. High-Performance Liquid Chromatography (HPLC) Analysis

The HPLC analysis of *C. okamurae* extract was conducted using a Waters 2690 XE separations module (Waters, Milford, MA, USA). Chromatographic separation was performed on a Waters SunFire C18 column (3.5 μm, 4.6 mm × 150 mm) maintained at 35 °C. The mobile phase consisted of 0.1% formic acid in distilled water (buffer A) and 0.1% formic acid in acetonitrile (buffer B). A linear gradient elution was employed over 35 min at a flow rate of 1 mL/min. The injection volume was set at 10 μL, and UV detection was carried out at 280 nm.

### 2.4. Cell Culture

The RAW 264.7 macrophage line (TIB-71, ATCC, Rockville, MD, USA) was maintained in Dulbecco’s Modified Eagle Medium (Gibco BRL, Carlsbad, CA, USA) supplemented with 10% fetal bovine serum (Gibco BRL) and 1% antibiotic-antimycotic solution (Gibco BRL). The cells were incubated in a humidified environment at 37 °C with 5% CO_2_. The cells were subcultured and plated at 80% confluency.

### 2.5. Cell Viability Assay

Cell viability was assessed using the 3-(4,5-dimethylthiazol-2-yl)-5-(3-carboxymethoxyphenyl)-2-(4-sulfophenyl)-2H-tetrazolium (MTS) assay (CellTiter 96^®^ AQueous One Solution Cell Proliferation Assay, Promega, Madison, WI, USA). This assay was performed following the manufacturer’s protocol, which relies on the reduction in MTS tetrazolium compound to formazan by the mitochondrial enzymes in viable cells. Briefly, RAW 264.7 macrophages were seeded into 96-well plates at a density of 1 × 10^5^ cells/well and incubated at 37 °C in a 5% CO_2_ atmosphere for 24 h. Cells were then treated with various concentrations of *C. okamurae* extract for an additional 24 h. Following treatment, MTS reagent was added to each well at a ratio of 1:5 and incubated for 2 h 30 min at 37 °C in a humidified 5% CO_2_ incubator. Absorbance was measured at 490 nm using a microplate reader (TECAN, Mänedorf, Zürich, Switzerland).

### 2.6. NO Assay

RAW 264.7 macrophages were seeded at a density of 5 × 10^5^ cells/well in 24-well plates and incubated overnight in a 5% CO_2_ incubator at 37 °C (Thermo Fisher Scientific, Rockford, IL, USA). The cells were pre-treated with various concentrations of *C. okamurae* extract for 2 h, followed by stimulation with 1 × 10^7^ colony forming units (CFU)/mL of *P. gingivalis* for 24 h. After incubation, 50 μL of cell culture supernatant was mixed with an equal volume of Griess reagent (Promega, Madison, WI, USA) and incubated at room temperature for 10 min. Nitric oxide (NO) production was quantified by measuring the absorbance at 540 nm using a microplate reader. A sodium nitrite (NaNO_2_) standard curve was used to calculate NO concentrations.

### 2.7. Quantitative Real-Time PCR (qRT-PCR)

RAW 264.7 macrophages were seeded into 24-well plates at a density of 5 × 10^5^ cells/well and incubated overnight at 37 °C in a humidified atmosphere containing 5% CO_2_. The cells were pretreated with varying concentrations of *C. okamurae* extract (0.2, 2, 20, and 200 μg/mL) for 2 h, followed by stimulation with *P. gingivalis* (1 × 10^7^ CFU/mL) for 24 h. Total RNA was extracted from the treated cells using TRIzol reagent (Ambion, Carlsbad, CA, USA) according to the manufacturer’s protocol. RNA concentration and purity were assessed using a Biospec-Nano spectrophotometer (Shimadzu, Nakagyo-ku, Kyoto, Japan). cDNA was synthesized from the isolated RNA using the PrimeScript RT Reagent Kit (TaKaRa, Shiga, Japan) in accordance with the manufacturer’s instructions. qRT-PCR was conducted using Power SYBR^®^ Green PCR Master Mix (Applied Biosystems, Warrington, UK) on a StepOnePlus Real-Time PCR (Applied Biosystems, Foster City, CA, USA). The thermal cycling conditions were set as follows: an initial denaturation step at 95 °C for 10 min, followed by 40 cycles of 95 °C for 15 sec and 60 °C for 1 min. Cycle threshold values were calculated using generated PCR curves and *β-actin* mRNA expression was used as a loading control to normalize the expression of *IL-1β*, *IL-6*, and *TNF-α* mRNA. The following primers were used for qRT-PCR—*IL-1β*: forward 5′-GAAAGACGGCACACCCACCCT-3′ and reverse 5′-GCTCTGCTTGTGAGGTGCTGATGTA-3′ (NM_008361); *IL-6*: forward 5′-GATGGATGCTACCAAACTGGA-3′ and reverse 5′-TCTGAAGGACTCTGGCTTTG-3′ (NM_031168); *TNF-α*: forward 5′-CCACCACGCTCTTCTGTCTAC-3′ and reverse 5′-AGGGTCTGGGCCATAGAACT-3′ (BC137720); and *β-actin*: forward 5′-CATCACTATTGGCAACGAGC-3′ and reverse 5′-GACAGCACTGTGTTGGCATA-3′ (NM_0073 93).

### 2.8. Western Blot Analysis

RAW 264.7 macrophages were incubated in 6-well plates containing different concentrations of *C. okamurae* extract (0.2, 2, 20, and 200 μg/mL) with *P. gingivalis* (1 × 10^7^ CFU/mL) for 24 h. The cells were harvested and washed twice with cold phosphate-buffered saline (Gibco, Grand Island, NY, USA). Cytoplasm and nucleus were separated using the Nuclear Extraction Kit (Cayman, Ann Arbor, MI, USA) and then protein quantification was performed using the Pierce™ BCA Protein Assay Kit (Thermo Fisher Scientific, Rockford, IL, USA). Quantified proteins from each lysate were separated by 10% SDS-PAGE and electro-transferred onto polyvinylidene fluoride membranes (Amersham Biosciences, Piscataway, NJ, USA). Membranes were blocked with 5% skim milk for 1 h at room temperature and washed three times with Tris-buffered saline containing Tween 20 (TBS-T). The membranes were then incubated with primary antibodies. The primary antibody dilutions used were as follows: iNOS (1:1000), NF-κB p65 subunit (1:1000), p-IκBα (1:1000), IκBα (1:1000), p-IKKα/β (1:1000), and β-actin (1:5000). After washing with TBST, the membranes were incubated with HRP-conjugated secondary antibodies for 1 h at room temperature. Protein bands were visualized using Immobilon Western Chemiluminescent HRP Substrate (Millipore, Billerica, MA, USA) and imaged with the cSeries Capture Software version 2.1.4.0731 (Azure Biosystems, Dublin, CA, USA). The detected bands were quantified using ImageJ 1.52a software (NIH, Bethesda, MD, USA).

### 2.9. Immunofluorescence Analysis

RAW 264.7 macrophages were seeded in 12-well plates at a density of 1 × 10^6^ cells/well. The cells were pre-treated with varying concentrations of *C. okamurae* extract, followed by *P. gingivalis* (1 × 10^7^ CFU/mL) stimulation. After PBS washing, cells were fixed with 4% paraformaldehyde for 15 min at room temperature. Fixed cells were washed three times with 1× Wash Buffer, with each wash lasting 5 min. Cells were blocked using Blocking Buffer for 1 h at room temperature. Subsequently, the cells were incubated overnight at 4 °C with primary antibodies against NF-κB p65 (1:1500 dilution), iNOS (1:250 dilution) and PI (1:1000 dilution). Following primary antibody incubation, cells were washed three times with 1× Wash Buffer for 5 min each. Secondary antibody (anti-rabbit IgG, 1:1700 dilution) was then added, and the cells were incubated for 1 h at room temperature in the dark. After three more 5 min washes with 1× Wash Buffer, the cells were mounted using ProLong^®^ Gold Antifade reagent (Cell Signaling Technologies, Danvers, MA, USA). Immunofluorescence images were captured using a Zeiss LSM 980 Confocal Microscope (Zeiss, Oberkochen, Germany).

### 2.10. Statistical Analysis

Statistical analyses were carried out using SPSS software version 25.0 (SPSS, Chicago, IL, USA). The experiments were repeated at least three times, and statistical significance was determined using Student’s *t*-test. Data are presented as the mean ± standard deviation (SD). A *p*-value < 0.05 was considered statistically significant.

## 3. Results

### 3.1. HPLC Analysis of C. okamurae Extract

**Figure 1:**
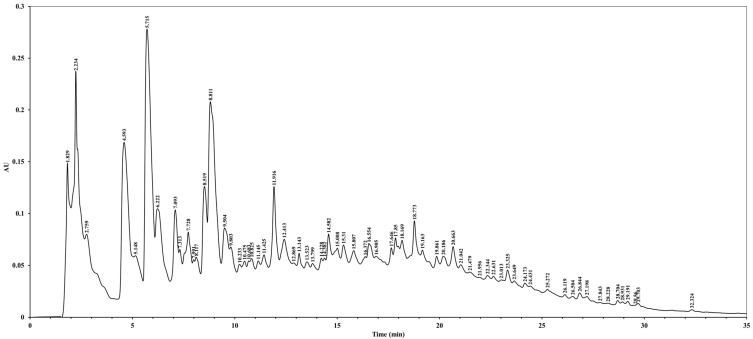
HPLC chromatogram of the crude extract of *Caulerpa okamurae* (*C. okamurae*). HPLC analysis of the crude *C. okamurae* extract was performed using a SunFire C18 column (3.5 μm, 4.6 mm × 150 mm) maintained at 35 °C. The analysis was conducted at a flow rate of 1 mL/min with the detection wavelength set at 280 nm.

To demonstrate the complexity of the *C. okamurae* extract and justify the use of the crude extract over isolated compounds, a full-scan HPLC analysis was performed. The HPLC chromatogram of the crude extract of *C. okamurae* revealed a total of 68 peaks, representing various bioactive compounds with different retention times (Figure 1). The major peak, with an area value of 5,848,326, was observed at 5.715 min, accounting for 7.38% of the total peak area. Other significant peaks were detected at 8.811 min (6.59%), 2.234 min (6.37%), and 4.593 min (5.42%). The retention times ranged from 1.829 to 32.324 min. The complexity of the chromatographic profile suggests the presence of numerous bioactive compounds in the *C. okamurae* extract, indicating potential synergistic interactions among these components.

### 3.2. RAW 264.7 Macrophage Cell Viability for C. okamurae Extract

**Figure 2:**
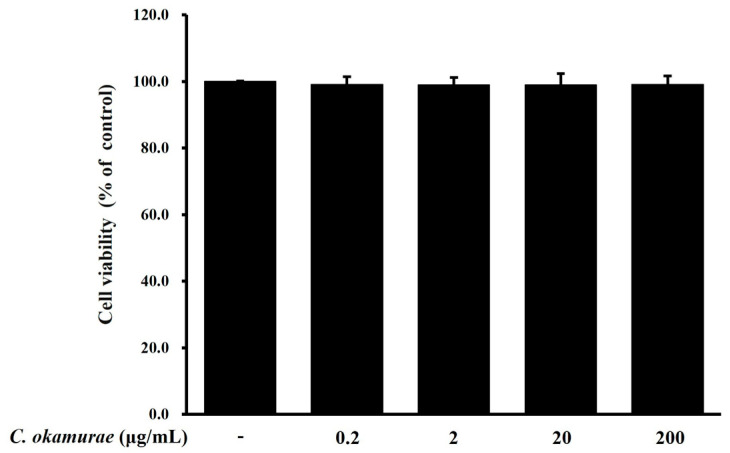
Effect of *C. okamurae* extract on the cell viability of RAW 264.7 macrophages. RAW 264.7 macrophages were treated with different concentrations of *C. okamurae* extract (0.2, 2, 20, and 200 μg/mL) for 24 h, following which cell viability was determined by an MTS assay. Data are presented as the mean ± SD (*n* = 3).

To investigate the effect of *C. okamurae* extract on the cell viability of RAW 264.7 macrophages, MTS assay was performed. RAW 264.7 macrophages were cultured for 24 h after treatment with various concentrations (0.2, 2, 20, 200 μg/mL) of *C. okamurae* extract. The results showed that the treatment with various concentrations did not affect the cell viability (Figure 2). Therefore, the experiment was conducted using the above concentration in subsequent experiments.

### 3.3. Effects of C. okamurae Extract on NO Production and iNOS Expression in P. gingivalis-Stimulated RAW 264.7 Macrophages

**Figure 3:**
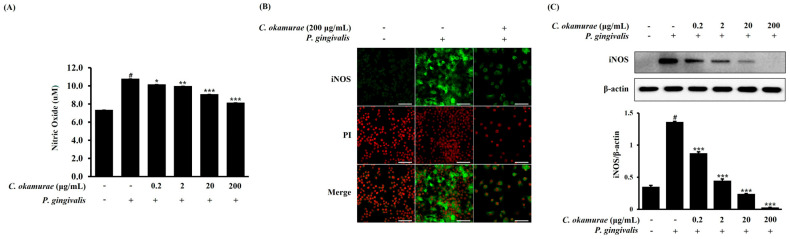
Effect of *C. okamurae* extract on NO production and inhibition of iNOS expression in RAW 264.7 macrophages stimulated with *P. gingivalis*. RAW 264.7 macrophages were treated with *C. okamurae* extract (0.2, 2, 20, and 200 μg/mL) and stimulated with *P. gingivalis* for 24 h. (**A**) NO concentration was measured using the Griess reaction. (**B**) Immunofluorescence staining with an iNOS antibody (green) was visualized using confocal microscopy. Nuclei were stained with PI (red). Scale bar = 50 μm. (**C**) Western blot analysis was performed on the prepared proteins. Image analysis of iNOS expression was performed using ImageJ. Data are presented as means ± SD of triplicates (# *p* < 0.05 compared with untreated control group; * *p* < 0.05, ** *p* < 0.01, *** *p* < 0.001 compared with *P. gingivalis*-treatment group).

To examine the effect of *C. okamurae* extract on NO production and iNOS expression in *P. gingivalis*-stimulated RAW 264.7 macrophages, cells were treated with *C. okamurae* extract (0.2, 2, 20, 200 μg/mL) and then stimulated with *P. gingivalis* for 24 h. When stimulated with *P. gingivalis*, NO production was significantly increased compared to the control group. In contrast, NO production was dose-dependently decreased following treatment with *C. okamurae* extract after stimulation with *P. gingivalis* (Figure 3A). iNOS, which catalyzes the production of NO, is actively expressed during inflammation. Western blot analysis demonstrated a marked increase in iNOS protein levels in *P. gingivalis*-stimulated RAW 264.7 macrophages. Notably, *C. okamurae* extract inhibited iNOS protein expression in a dose-dependent manner (Figure 3B). The confocal microscopy analysis indicated that *P. gingivalis* stimulation significantly enhanced iNOS expression in the cytoplasm, while treatment with *C. okamurae* extract resulted in a substantial decrease in its cytoplasmic levels (Figure 3C).

### 3.4. Effect of C. okamurae Extract on Expression of Proinflammatory Cytokines in RAW 264.7 Macrophages Stimulated with P. gingivalis

**Figure 4:**
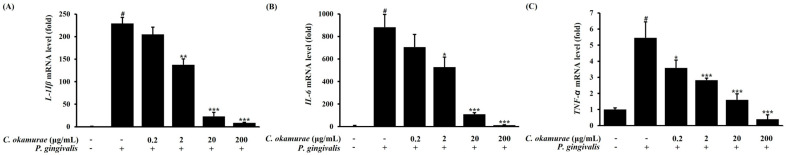
Effect of *C. okamurae* extract on the expression of proinflammatory cytokines in RAW 264.7 macrophages stimulated with *P. gingivalis*. RAW 264.7 macrophages were pretreated with *C. okamurae* extract (0.2, 2, 20, and 200 μg/mL) and then stimulated with *P. gingivalis* for 24 h. (**A**) *IL-1β*, (**B**) *IL-6*, and (**C**) *TNF-α* mRNA levels were measured by qRT-PCR. mRNA levels were normalized to *β-actin*. Data are presented as means ± SD of triplicates (# *p* < 0.05 compared with untreated control group; * *p* < 0.05, ** *p* < 0.01, *** *p* < 0.001 compared with *P. gingivalis*-treatment group).

To assess whether treatment with *C. okamurae* extract inhibits the production of proinflammatory cytokines (*IL-1β*, *IL-6*, and *TNF-α*) in *P. gingivalis*-stimulated RAW 264.7 macrophages, qRT-PCR was performed. In the *P. gingivalis*-treated group, mRNA levels of *IL-1β*, *IL-6*, and *TNF-α* were markedly increased. Conversely, *C. okamurae* extract treatment significantly reduced the mRNA expression of these proinflammatory cytokines in a concentration-dependent manner (Figure 4A–C).

### 3.5. Effect of C. okamurae Extract on the Nuclear Translocation of NF-κB p65 in RAW 264.7 Macrophages Stimulated with P. gingivalis

**Figure 5:**
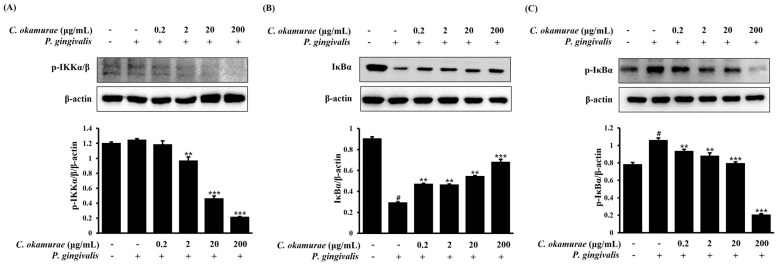
Effect of *C. okamurae* extract on p-IKKα/β and p-IκBα expression in RAW 264.7 macrophages stimulated with *P. gingivalis*. RAW 264.7 macrophages were treated with *C. okamurae* extract (0.2, 2, 20, and 200 μg/mL) and then stimulated with *P. gingivalis* for (**A**) 5 min and (**B**,**C**) 20 min. Western blot analysis was performed on the prepared proteins. Image analysis of each protein expression was performed using ImageJ. Data are presented as means ± SD of triplicates (# *p* < 0.05 compared with untreated control group; ** *p* < 0.01, *** *p* < 0.001 compared with *P. gingivalis*-treatment group).

**Figure 6:**
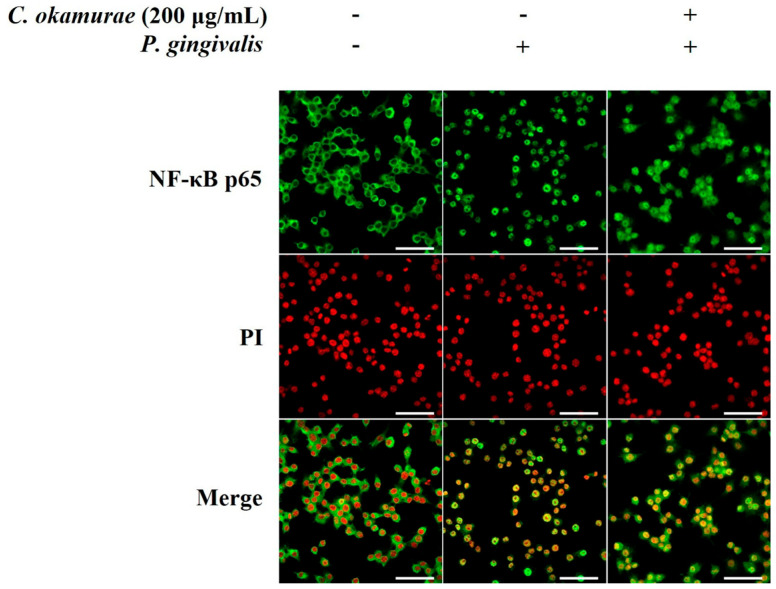
Effect of *C. okamurae* extract on *P. gingivalis*-stimulated NF-κB p65 translocation in RAW 264.7 macrophages. RAW 264.7 macrophages were treated with *C. okamurae* extract and stimulated with *P. gingivalis* for 30 min. Confocal microscopy was performed after immunofluorescence staining. NF-κB p65 (green) and cell nuclei (red, PI staining) were visualized (Scale bar = 50 μm).

NF-κB is a representative inflammatory signaling pathway and is activated in stimulated cells and is involved in the transcriptional activation of inflammatory genes. The effect of *C. okamurae* extract on NF-κB pathway activation in *P. gingivalis*-stimulated RAW 264.7 macrophages was investigated by Western blot and immunofluorescence. RAW 264.7 macrophages were treated with *C. okamurae* extract (0.2, 2, 20, and 200 μg/mL) and then stimulated with *P. gingivalis*. Phosphorylation of IKKα/β, IκBα was analyzed by Western blot. In RAW 264.7 macrophages stimulated by *P. gingivalis*, treatment with *C. okamurae* extract inhibited the phosphorylation of IKKα/β and IκBα, resulting in decreased protein expression (Figure 5). The results of analyzing NF-κB p65 translocation by immunofluorescence revealed that the *C. okamurae* extract had an inhibitory effect on translocation into the nucleus (Figure 6).

## 4. Discussion

Recently, various extracts derived from marine resources have been of increasing interest for therapeutic applications. Previous studies on *C. okamurae* have mainly focused on its antidiabetic and anti-obesity effects [32,33], but studies on its anti-inflammatory effects have not been thoroughly examined. Therefore, in this study, we investigated its anti-inflammatory effects in RAW 264.7 macrophages stimulated with *P. gingivalis*, which is the periodontal pathogen. This is the first study to elucidate the anti-inflammatory effects of *C. okamurae* after infection with a periodontal pathogen.

We evaluated whether *C. okamurae* extract could suppress the inflammatory response induced by *P. gingivalis* in RAW 264.7 macrophages. The inflammatory response induced by *P. gingivalis* promotes the expression of proinflammatory cytokines along with NO production and iNOS expression in RAW 264.7 macrophages. *C. okamurae* extract reduced the expression of these inflammatory mediators. To rule out the possibility that cytotoxicity from *C. okamurae* extract treatment might contribute to the inhibition of inflammatory mediators, cell viability was tested after treatment with various concentrations (0.2, 2, 20, and 200 μg/mL) of *C. okamurae* extract. No cytotoxicity was detected at any concentration used. NO is an important signaling molecule in inflammatory responses, and when produced excessively, it can amplify inflammation by inducing DNA damage, cell death, and ROS accumulation. NO produced by iNOS is essential for pathogen defense, but excessive production can cause tissue damage and dysfunction of the immune system. Therefore, regulating iNOS expression can be evaluated as an important strategy for the development of inflammatory disease treatments. In this study, *P. gingivalis* stimulation of RAW 264.7 macrophages increased NO production, but NO production in these macrophages was reduced by *C. okamurae* extract treatment. Furthermore, it was found that iNOS protein expression was significantly increased after *P. gingivalis* stimulation, but iNOS expression was decreased in a dose-dependent manner when *C. okamurae* extract was treated. These results suggested that *C. okamurae* extract has an anti-inflammatory effect.

*P. gingivalis* LPS is known to be one of the major causes of periodontitis and plays an important role in inflammatory response and alveolar bone loss. It also induces NO production in macrophages and gingival fibroblasts and acts as a mediator of inflammatory response [4,5,6,7]. This study used *P. gingivalis* as a stimulant and showed that *C. okamurae* extract inhibited iNOS expression and reduced *P. gingivalis*-induced NO production in RAW 264.7 macrophages.

LPS activates the NF-κB signaling pathway to regulate gene expression and amplify inflammatory response. NF-κB is activated by the degradation of IκB protein, and this process is regulated by the action of the IκB kinase (IKK) complex. LPS transmits signals through its receptor to sequentially activate the IKK, IκBα, and NF-κB signaling pathways. In response, NF-κB translocates to the nucleus and increases the transcription of inflammatory mediator genes, ultimately producing inflammatory cytokines such as *IL-1β*, *IL-6*, and *TNF-α*. In this process, the expression of COX-2, iNOS, and various chemokines and cytokines is promoted to maintain and amplify the inflammatory response [16,17,18,19,20,21]. Excessive activation of this NF-κB signaling causes tissue damage, fibrosis, and metabolic disorders, and promotes the pathological mechanisms of various chronic diseases. Therefore, regulation of the NF-κB pathway is considered an important strategy for the treatment of inflammatory chronic diseases. In this study, we evaluated whether the expression of *IL-1β*, *IL-6*, and *TNF-α* mRNA was reduced by treatment with *C. okamurae* extract in RAW 264.7 macrophages stimulated with *P. gingivalis* using qRT-PCR. In RAW 264.7 macrophages stimulated with *P. gingivalis*, the expression of *IL-1β*, *IL-6*, and *TNF-α* was significantly increased, while mRNA expression was decreased by *C. okamurae* extract treatment. Interestingly, IκBα phosphorylation and IKKα/β phosphorylation were decreased after *C. okamurae* extract treatment, and NF-κB nuclear translocation was inhibited.

Research on the discovery of anti-inflammatory substances using marine organisms began relatively late but is progressing rapidly. The marine environment is rich in biodiversity and unique biological groups, and marine organisms contain many potential biologically active compounds. Previous studies have shown that marine actinomycetes produce various anti-inflammatory compounds, and marine cyanobacteria produce peptides and polyketides that can control inflammatory responses. Fucoidan—a sulfated polysaccharide extracted from brown seaweed—has been shown to significantly inhibit LPS-induced NO production and proinflammatory cytokine expression in macrophages by downregulating the NF-κB and MAPK pathways. Similarly, carrageenan from red algae is known to exhibit immunomodulatory effects by reducing the release of TNF-α and IL-6. In addition, omega-3 fatty acids found in marine vertebrates and fish, spongiolides found in marine invertebrates, and dolastatin extracted from marine snails and mollusks have been reported to have potent anti-inflammatory effects [35]. In our study, *C. okamurae* extract also suppressed NO production and iNOS expression in a dose-dependent manner, suggesting a comparable mechanism of action. In contrast to pharmaceutical agents such as dexamethasone, which exerts a broad and potent anti-inflammatory effect but is associated with systemic side effects, *C. okamurae* extract did not exhibit cytotoxicity at any of the tested concentrations, indicating its potential as a safer natural alternative [36].

The results of this study demonstrate the anti-inflammatory effects of *C. okamurae* extract and suggest the usefulness of preventive *C. okamurae* extract.

## 5. Conclusions

In conclusion, this study suggests that *C. okamurae* extract exhibits anti-inflammatory effects. *C. okamurae* extract suppressed inflammatory signaling in RAW 264.7 macrophages stimulated with *P. gingivalis* by reducing IKK and IκBα phosphorylation, inhibiting NF-κB activation, and blocking NF-κB nuclear translocation. In addition, it reduced the expression of iNOS, *IL-1β*, *IL-6*, and *TNF-α*. Although further in vivo studies are needed to confirm the anti-inflammatory effects of *C. okamurae* extract, these results indicate the molecular mechanism underlying its anti-inflammatory effects of *C. okamurae* extract. Overall, these findings suggest that *C. okamurae* extract may be used in the development of therapeutic agents to help prevent periodontal disease.

## Data Availability

The original contributions presented in the study are included in the article; further inquiries can be directed to the corresponding authors.
